# Supplementary material for: Functional dissection of Wag31 domains for septal recruitment and polar distribution during the cell cycle
Source: bioRxiv. 2025 Aug 21:2025.08.21.671543. Preprint. [Version 1] doi: 10.1101/2025.08.21.671543 (PMC12393619; doi:10.1101/2025.08.21.671543)
Supplement: Supplement 1 [file media-1.pdf]

Supplementary Information for:

Functional dissection of Wag31 domains for septal recruitment and polar distribution.

Julienne Petit[1,2], Daniela Megrian[1*], Mariano Martinez[1,2], Adrià Sogues[1], Célia De Sousa-D'Auria[3], Mathilde Ben Assaya[1], Catherine Thouvenot[4], Emilie Lesur[5], Yann Bourdreux[5], Nicolas Bayan[3], Pedro M Alzari[2] & Anne Marie Wehenkel[1#].

1. Institut Pasteur, Université Paris Cité, CNRS UMR 3528, Bacterial Cell Cycle Mechanisms Unit, F-75015 Paris, France
2. Institut Pasteur, Université Paris Cité, CNRS UMR 3528, Structural Microbiology Unit, F-75015 Paris, France
3. Université Paris-Saclay, CEA, CNRS, Institute for Integrative Biology of the Cell (I2BC), 91198, Gif-sur-Yvette, France.
4. Institut Pasteur, Université Paris Cité, Ultrastructural BioImaging Unit, F-75015 Paris, France
5. Université Paris-Saclay, CNRS UMR 8182, Institut de Chimie Moléculaire et des Matériaux d'Orsay, ICMMO, F-91405 Orsay, France.

*Current address: Institut Pasteur de Montevideo, Bioinformatics Unit, 11200 Montevideo, Uruguay.

[#]Correspondance: anne-marie.wehenkel@pasteur.fr

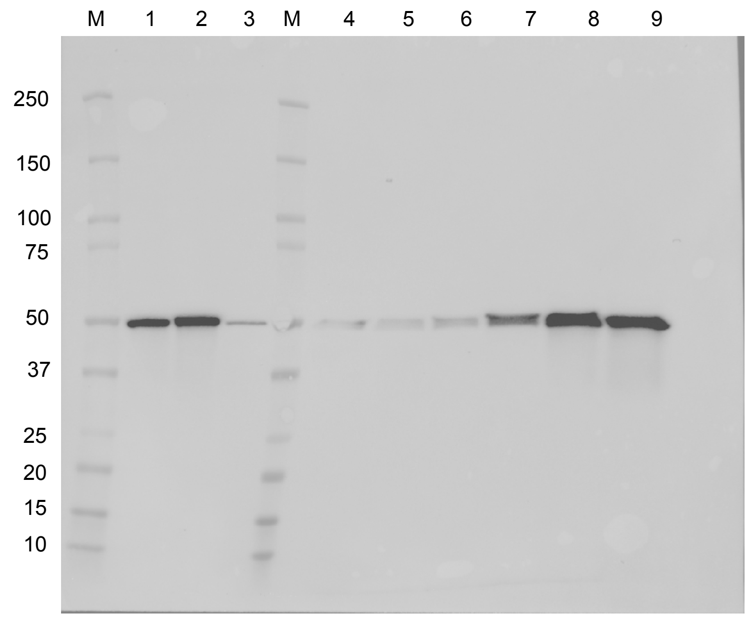

Supplementary Figure S1. *Cglu*_P$_{ino}$-*wag31* complementation. Western blot of whole cell extracts from *Cglu* (1), *Cglu* + empty plasmid (2) and *Cglu*_P$_{ino}$-*wag31* (3) after overnight growth in minimal medium. Depleted *Cglu*_P$_{ino}$-*wag31* complemented with P$_{gntk}$-wag31 plasmid in 4% sucrose after 0 (4) and 3 (5) and 6 (6) hours and in the presentce of 1% gluconate (overexpression) after 0 (7) and 3 (8) and 6 (9) hours. Wag31 levels were revealed using anti-Wag31$_{1\text{-}61}$ antibody. Lanes M contain molecular weight markers.

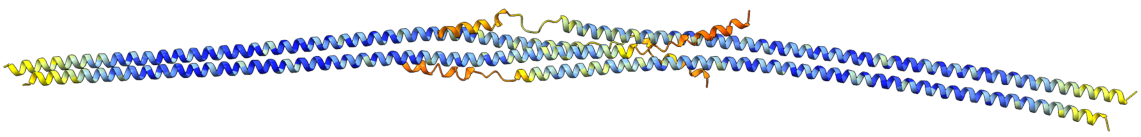

Supplementary Figure S2. AF model of the C-terminal tetramerization interface of Wag31.

Table S1. Bacterial strains, plasmids and primers used in this study, with descriptions and references.

**Strains**	**Characteristics**	**Reference**
E. coli		
DH5*α*	F- endA1 Φ80dlacZΔM15 Δ(lacZYA-argF)U169 recA1 relA1 hsdR17(rK–mK+) deoR supE44 thi-1 gyrA96 phoA λ–; strain used for general cloning procedures	[1]
CopyCutter EPI400	F- mcrA Δ(mrr-hsdRMS-mcrBC) Φ80dlacZΔM15 ΔlacX74 recA1 endA1 araD139 Δ(ara, leu)7697 galU galK λ- rpsL (StrR) nupG trfA tonA pcnB4 dhfr; strain used for general cloning procedures	[2]
BL21(DE)	F- ompT hsdSB(rB–mB–) gal dcm (DE3); host for protein production	[3]
C. glutamicum		
Cglu (ATCC 13032)	Biotin-auxotrophic wild type	[4]
Cglu_*P_{ino}-wag31* (sUMS_214)	*myo*-inositol dependent *wag31* silencing strain. ATCC 13032 with insertion of a terminator and P_{ino} promoter to silence *wag31* (*cg2361)* expression. Repressible in the presence of *myo*-inositol	This work

**Plasmids for *C. glutamicum wag31* conditional depletion**		**Reference**
pK19mobsacB	KanaR; plasmid for allelic exchange in *C. glutamicum*; (pK18 oriVEc, sacB, lacZα)	[5]
pk19-P3323-lcpA	KanaR; pK19mobsacB derivative. Used as a PCR template to amplify a transcriptional terminator and the promoter of cg3323 (P_{ino})	[6,7]
pK19-P_{ino}*-wag31*	KanaR; pk19mobsacB derivative containing 500 bp upstream-region of *wag31*, a transcriptional terminator, the P_{ino} promoter and 500bp of the *wag31* coding region	This work

**Plasmids for recombinant protein expression in *C. glutamicum*.**		**Reference**
pTGR5	KanaR; *E. coli/C. glutamicum* shuttle vector for regulated gene expression of EGFP under control of tac promoter (Ptac lacI ColE1 oriVEc pGA1 oriVCg)	[8]
pUMS_3	KanaR; pTGR5 derivative in which *Ptac* was exchanged by *PgntK* promoter to control the expression of the EGFP protein	[9]
pUMS_17	KanaR; pUMS3 derivative for expression of Wag31 under control of *PgntK* promoter	This work
pUMS_25	KanaR; pUMS3 derivative for expression of Wag31-mNeonGreen (C-terminal tag) under control of *PgntK* promoter	This work
pUMS_21	KanaR; pUMS3 derivative for expression of mNeonGreen-Wag31 (N-terminal tag) under control of *PgntK* promoter	This work
pUMS_175	KanaR; pUMS3 derivative for expression of $Wag31_{LBD}$ (residues 1-61) fused to mNeonGreen (C-terminal tag) under control of *PgntK* promoter	This work
pUMS_176	KanaR; pUMS3 derivative for expression of $Wag31_{CCD}$ (residues 62-365) fused to mNeonGreen (C-terminal tag) under control of *PgntK* promoter	This work
pUMS_254	KanaR; pUMS3 derivative for expression of $Wag31_{mut}$, carrying the single mutations I18D, K20S, R21S, fused to mNeonGreen (C-terminal tag) under control of *PgntK* promoter	This work

pUMS_255	KanaR; pUMS3 derivative for expression of Wag31$_{LBD_mut}$, carrying the single mutations I18D, K20S, R21S, fused to mNeonGreen (C-terminal tag) under control of *PgntK* promoter	This work

Oligonucleotide	Sequence 5' -->3' and properties[a]
**Plasmids for *C. glutamicum* strain construction**	
pK19-P$_{ino}$-wag31	
P7_JP	TGTTGTGTGGAATTGTGCCGCTAGGTAATGTGCGC
P8_JP	TTGCGGATTCCCTTCGATTTAACGG
P9_JP	GAAGGGAATCCGCAAATAAAACGAAAGGCTCAGTCGAAAGAC
P10_JP	TGGAGTCAACGGCATCTAAAATTTCTCCTCTTAAAAAGATAACGGCC
P11_JP	ATGCCGTTGACTCCAGCTGATGT
P12_JP	AATTGTTATCCGCTCAGTCCACATTTGCAGCACCTGTAGC

**Plasmids for recombinant protein expression in *C. glutamicum***	
pUMS_17	
P178_MM	ATGGTCTTATCCTTTCTTTGGTGGC
P179_MM	AGCGGCCGCTTAAGGTAC
P216_MM	CCAAAGAAAGGATAAGACCATATGCCGTTGACTCCAGCTGATGT
P218_MM	CGGTACCTTAAGCGGCCGCTTTACTCACCAGATGGCTTGTTG
pUMS_25	
P178_MM	ATGGTCTTATCCTTTCTTTGGTGGC
P179_MM	AGCGGCCGCTTAAGGTAC
P214_MM	CTCGAGGGATCTGGCCAGGGACCGGGCTCAGGCCAAGGAAGCGGCATGGTGTCCAAGGGCGAAGAG
P215_MM	GAATTCGGTACCTTAAGCGGCCGCTTTACTTGTACAGTTCATCCATGCCCATCACATCGGTGAATG
P216_MM	CCAAAGAAAGGATAAGACCATATGCCGTTGACTCCAGCTGATGT
P217_MM	CCCTGGCCAGATCCCTCGAGCTCACCAGATGGCTTGTTGTTG
pUMS_21	
P178_MM	ATGGTCTTATCCTTTCTTTGGTGGC
P179_MM	AGCGGCCGCTTAAGGTAC
P190_MM	CAAAGAAAGGATAAGACCATATGGTGTCCAAGGGCGAAG
P191_MM	GCCAGATCCCTCGAGCTTGTACAGTTCATCCATGCCC
P212_MM	ACAAGCTCGAGGGATCTGGCCAGGGACCGGGCTCAGGCCAAGGAAGCGGCATGCCGTTGACTCCAGCTGATGT
P218_MM	CGGTACCTTAAGCGGCCGCTTTACTCACCAGATGGCTTGTTG

pUMS_175	
P13_JP	GGCAACCTGCGCCTCTAGCTCT
P14_JP	GCGCAGGTTGCCCTCGAGGGATCTGGCCAGGGAC
pUMS_176	
P15_JP	CATATGGTCTTATCCTTTCTTTGGTGGCGT
P16_JP	TAAGACCATATGGGTGGTACTTCTTCCGCTGCTAGTT
pUMS_254 and pUMS_255	
P39_JP	CCTGACGGCTCCAGTGGCTACAACGAAG
P40_JP	CACTGGAGCCGTCAGGCGGCTTATTAAAAGC

[a]Overlaps for Gibson assembly or site-directed mutations are highlighted in red

Supplementary References

1. Hanahan, D. Studies on transformation of Escherichia coli with plasmids. *J Mol Biol* **166**, 557–580 (1983).

2. D, H. *Epicentre Forum* **11**, 6 (2004).

3. Studier, F. W. & Moffatt, B. A. Use of bacteriophage T7 RNA polymerase to direct selective high-level expression of cloned genes. *J Mol Biol* **189**, 113–130 (1986).

4. Shimono, S. K. S. U. M. Studies on the amino acid fermentation. *J Gen Appl Microbiol* **3**, (1957).

5. Schäfer, A. *et al.* Small mobilizable multi-purpose cloning vectors derived from the Escherichia coli plasmids pK18 and pK19: selection of defined deletions in the chromosome of Corynebacterium glutamicum. *Gene* **145**, 69–73 (1994).

6. Baumgart, M., Schubert, K., Bramkamp, M. & Frunzke, J. Impact of LytR-CpsA-Psr Proteins on Cell Wall Biosynthesis in Corynebacterium glutamicum. *J Bacteriol* **198**, 3045–3059 (2016).

7. Sogues, A. *et al.* Essential dynamic interdependence of FtsZ and SepF for Z-ring and septum formation in Corynebacterium glutamicum. *Nat. Commun.* **11**, 1641 (2020).

8. Ravasi, P., Peiru, S., Gramajo, H. & Menzella, H. G. Design and testing of a synthetic biology framework for genetic engineering of Corynebacterium glutamicum. *Microb Cell Fact* **11**, 1–1 (2012).

9. Sogues, A. *et al.* Essential dynamic interdependence of FtsZ and SepF for Z-ring and septum formation in Corynebacterium glutamicum. *Nat Commun* **11**, 1641 (2020).
